# Polymorphisms in *C-Reactive Protein* and *Glypican-5* Are Associated with Lung Cancer Risk and *Gartrokine-1* Influences Cisplatin-Based Chemotherapy Response in a Chinese Han Population

**DOI:** 10.1155/2015/824304

**Published:** 2015-04-27

**Authors:** Shuo Zhang, Asmitananda Thakur, Yiqian Liang, Ting Wang, Lei Gao, Tian Yang, Yang Li, Tingting Geng, Tianbo Jin, Tianjun Chen, Johnson J. Liu, Mingwei Chen

**Affiliations:** ^1^Department of Respiratory and Critical Care Medicine, The First Affiliated Hospital of School of Medicine of Xi'an Jiaotong University, Xi'an, Shaanxi 710061, China; ^2^National Engineering Research Center for Miniaturized Detection Systems, School of Life Sciences, Northwest University, Xi'an 710069, China; ^3^School of Pharmacy, Faculty of Health Science, University of Tasmania, Hobart, TAS 7001, Australia

## Abstract

The role of genetics in progression of cancer is an established fact, and susceptibility risk and difference in outcome to chemotherapy may be caused by the variation in low-penetrance alleles of risk genes. We selected seven genes (*CRP, GPC5, ACTA2, AGPHD1, SEC14L5, RBMS3*, and* GKN1*) that previously reported link to lung cancer (LC) and genotyped single nucleotide polymorphisms (SNPs) of these genes in a case-control study. A protective allele “C” was found in rs2808630 of the C-reactive protein (*CRP*). Model association analysis found genotypes “T/C” and “C/C” in the dominant model and genotype “T/C” in the overdominant model of rs2808630 associated with reduced LC risk. Gender-specific analysis in each model showed that genotypes “T/T” and “C/C” in rs2352028 of the* Glypican 5* (*GPC5*) were associated with increased LC risk in males. Logistic regression analysis showed “C/T” genotype carriers of rs4254535 in the* Gastrokine 1* (*GKN1*) had less likelihood to have chemotherapy response. Our results suggest a potential association between* CRP* and* GPC5* variants with LC risk; variation in* GKN1* is associated with chemotherapy response in the Chinese Han population.

## 1. Introduction

Lung cancer is the most common malignancy in the world and is reported to have an increasing incidence in developing countries [1, 2]. According to the global cancer statistics, in 2008 approximately 1.6 million people were diagnosed with lung cancer, and there were 1.4 million deaths [3]. Tobacco smoke, environmental pollution, occupational exposures, and preexisting lung disease increase the risk of lung cancer. However, patients have been diagnosed with lung cancer in the absence of these risk factors [4–6]. Genetic susceptibility to lung cancer independent of established risk factors has not yet been clearly defined.

Despite considerable advances in the field of tumor biology, the majority of patients with lung cancer are diagnosed at an already advanced stage and thus surgical resection is not a feasible treatment option. Platinum-based doublet chemotherapy is the current standard of therapy in this situation. However, the response to chemotherapy among lung cancer patients has significant variation. We hypothesize that the susceptibility risk and variation in outcome to chemotherapy may be caused by the variation in low-penetrance alleles.

In this study, we selected single nucleotide polymorphisms (SNPs) from seven different genes* (CRP (C-reactive protein), GPC5 (Glypican 5), ACTA2 (actin, alpha 2, smooth muscle, aorta), AGPHD1 (aminoglycoside phosphotransferase domain containing 1), SEC14L5 (SEC14-like 5), RBMS3 (RNA binding motif, single stranded interacting protein 3), and GKN1 (Gastrokine 1))* that have been linked to lung cancer [7–13]. We analyzed each tag single nucleotide polymorphism (tSNP) for lung cancer risk in a case-control study involving Chinese population. Multivariate logistic regression analysis was used to test the association between gene polymorphisms and chemotherapy response.

## 2. Materials and Methods

### 2.1. Study Participants

A case-control study involving the Chinese study population of 309 lung cancer patients and 310 controls was conducted at the First Affiliated Hospital of Xi'an Jiaotong University. All included patients had recently diagnosed and histopathologically confirmed primary lung cancer. The control subjects were recruited from the health check-up center of the First Affiliated Hospital of Xi'an Jiaotong University, which they had visited for an annual health examination. Patients were ascertained to be free from any acute or chronic pathology. Their cancer-free status was reconfirmed by testing for plasma levels of carcinoembryonic antigen and alpha-fetoprotein. Blood samples from the patients were collected before initiation of chemotherapy or radiotherapy. Demographic and related clinical data of the study population was collected by a face-to-face questionnaire and medical case record. Patients were categorized as smokers or nonsmokers. The smokers were defined as those who smoked one cigarette/pipe per day for twelve months or longer at any time in their life. All of the participants were genetically unrelated ethnic Han Chinese from Shaanxi Province and provided written informed consent for their participation in the present study. The protocols for this study were conducted according to the Declaration of Helsinki and were approved by the Institutional Review Boards of both the First Affiliated Hospital of Xi'an Jiaotong University and Northwest University.

Five milliliters of whole blood were collected from each subject into tubes containing ethylenediaminetetraacetic acid (EDTA) at the time of initial diagnosis. After centrifugation, the samples were stored at −80°C until further use.

### 2.2. Evaluation of Cisplatin-Based Chemotherapeutic Response

There are all together 113 lung cancer patients who received cisplatin based first-line chemotherapy and satisfied the following criteria: Eastern Cooperative Oncology Group (ECOG) performance status (PS) ≤ 1, age > 18 years, and adequate bone marrow reserve, as well as satisfactory liver and renal function. These patients were in clinical stage III or IV and had a measurable lesion on computed tomography scan at the beginning of treatment. The patients received chemotherapy every 3 weeks, for a maximum of six cycles or until disease progression or unacceptable toxicity occurred. Response to treatment was determined according to the Response Evaluation Criteria in Solid Tumor Group (RECIST) guidelines after two cycles of chemotherapy and every two cycles thereafter [14]. For data analysis, patients achieving complete response (CR) or partial response (PR) were considered “responders,” and patients with stable disease (SD) or progressive disease (PD) were defined as “nonresponders” [15]. Multivariate logistic regression analysis was used to test the association between gene polymorphisms and chemotherapy response.

### 2.3. tSNP Selection and Genotyping

All seven tSNPs in the selected genes were associated with lung cancer and with minor allele frequencies (MAF) greater than 5% in the HapMap CHB (Chinese Han Beijing) population. DNA was extracted from whole blood by GoldMag-Mini Whole Blood Genomic DNA Purification Kit (GoldMag Co., Ltd., Xi'an City, China). The concentration was measured by NanoDrop 2000 (Thermo Scientific, Waltham, Massachusetts, USA). The design of primers, SNP genotyping, and data processing were performed by Sequenom MassARRAY platform Software (Sequenom Co., Ltd., San Diego, California, USA) [16, 17].

### 2.4. Statistical Analysis

Statistical analysis was undertaken using statistical software (SPSS 16.0; Chicago, IL) and Microsoft Excel. A two-sided *P* value < 0.05 was considered the threshold for statistical significance. Hardy-Weinberg equilibrium (HWE) of each tSNP in control group was tested by Fisher's exact test. The differences in allelic frequencies between case and control groups were compared via the Chi-squared test [18]. Associations between genotypes and lung cancer risk were tested in different genetic models (codominant, dominant, recessive, overdominant, and log-additive) by SNPStats website software http://bioinfo.iconcologia.net/snpstats/start.htm [19]. Testing of odds ratios (ORs) with 95% confidence intervals (CIs) was performed by unconditional logistic regression analysis with adjustment for gender and age [20]. Akaike's Information Criterion and Bayesian Information Criterion were applied to estimate the best-fit model for each SNP. Association between genotypes and lung cancer risk was determined by SNPStats for gender-specific populations under each model [19].

## 3. Results

We recruited 309 patients (74 females and 235 males, mean age at diagnosis 58 years, range 25–85, SD ± 10) and 310 healthy (113 females and 197 males, mean age at diagnosis 50 years, range 29–75, SD ± 8) individuals into our study (Table 1). The genotype profiles of our study patients are shown in Supplementary Table S1 in the Supplementary Material available online at http://dx.doi.org/10.1155/2015/824304. The SNPs and primers used in the multiplexed SNP MassEXTENED assay are presented in Table 2. None of the tSNPs that we evaluated among the control group deviated from HWE (Table 3). We hypothesized that the minor allele of each SNP was a risk factor compared with the wild-type allele.

A significant protective allele “C” was found in rs2808630 of the* CRP* gene based on the crude *P* value of 0.05 (OR = 0.66; 95% CI, 0.48–0.91; *P* = 0.01) by Chi-square test (Table 3). Various genetic models were applied to calculate genetic risk. Reduced risk for lung cancer was associated with the genotypes “T/C” and “C/C” in rs2808630 (OR = 0.66, 95% CI, 0.44–0.98; *P* = 0.036) in the dominant model and the genotype “T/C” (OR = 0.65, 95% CI, 0.43–0.98; *P* = 0.037) in the overdominant model (Table 4). Each tSNP was analyzed in a gender-specific population under each model. We found that the genotypes “T/T” and “C/C” in rs2352028 of the* GPC5* gene were associated with increased lung cancer risk in males in the overdominant model (Table 5). rs2808630 in* CRP* and rs2352028 in* GPC5* were both associated with lung cancer risk.

“C/T” genotype distribution in the rs4254535 of the* GKN1* gene was significantly higher in nonresponders than in responders (34.62% versus 14.29%, *P* = 0.029) (Table 6). Logistic regression analysis showed that “C/T” genotype carriers had poor response for chemotherapy as compared to “T/T” genotype carriers (OR 3.287, 95% CI, 1.135–9.522; *P* = 0.029) after adjustment for age, gender, smoking status, histology, stage, and chemotherapy regimens.

However, as shown in Tables 3, 4, and 6, the significance levels were attenuated after applying a strict Bonferroni correction, indicating a likely association between positive tSNPs and risk of lung cancer and chemotherapy response.

## 4. Discussion

In this case-control study, we selected tSNPs with MAF greater than 5% in the HapMap CHB population to ensure that the statistical power was sufficient for data analysis. Our results firstly suggest that polymorphisms in* CRP* and* GPC5 *genes have an association with susceptibility risk of lung cancer in the Chinese Han population. The multivariate logistic regression analysis shows that polymorphism in* GKN1 *influences chemotherapy response.

The* CRP* gene, located in 1q23.2, encodes CRP protein which has several host defense-related functions, including recognition and elimination of foreign pathogens and damaged host cell. CRP is an acute-phase protein that increases during the host response to tissue injuries, including infection, trauma, surgery, myocardial infarct, and cancer [8, 21]. There are three potential mechanisms linking CRP to cancers. One is that tumor growth promotes tissue inflammation and increases the level of CRP. Another possibility is that cancer cells increase production of inflammatory proteins, which leads to high CRP levels in cancer patients. Besides, CRP may promote tumor growth in chronic inflammation [22]. Elevated CRP levels are associated with poor prognosis of lung, hepatic, renal, colorectal, and ovarian cancers [23–29].

Our study found that rs2808630, an intronic SNP within the* CRP* gene, was significantly linked with lung cancer risk in both allelic and genotypic association analysis of a Chinese population. We also ascertained a significant allele “C” and genotypes “T/C” and “C/C” in rs2808630 in the dominant model and genotype “T/C” in the overdominant model that is protective against lung cancer development. We hypothesize that rs2808630 variant of the* CRP* gene could have decreased the level of CRP or reduced the activity of CRP in the presence of allele “C”. A recent study by Xu et al. [30] found that 5 SNPs in the* CRP* gene (including rs2808630) were uncorrelated with lung cancer risk. They recruited 96 lung cancer patients and 124 controls of different races. This disparity in findings could be attributed to the small sample size and racial or regional differences in study populations. To our knowledge, our study is the first genotype/allele-based study that describes the association between SNPs within the* CRP* locus and lung cancer risk in a Chinese population.

The* GPC5* gene is a member of the glypican gene family and has eight exons encoding 572 amino acids in a large genomic region (1.47 Mb) of chromosome 13q31.3. Reduction of GPC5 protein is linked to lung cancer [7]. A previous study involving American population reported an association (OR = 1.46, 95% CI 1.26–1.70, *P* = 5.94 × 10^−6^) between the single nucleotide polymorphism rs2352028 and lung cancer risk in never smokers [31] but failed to replicate in Caucasian [32] and Chinese [33] populations, indicating that the sensitivity and specificity of rs2352028 in terms of smoking status may not be similar in between races. Our study observed the variation between gender and found that genotypes “T/T” and “C/C” in rs2352028 of the* GPC5* gene are associated with increased lung cancer risk in males (under the overdominant model, after adjusting for age).

The* GKN1 *gene is located in 2p13.3 and has a protective function on gastric antral mucosa by facilitating restoration and proliferation after injury. As it is expressed in normal gastric tissue but absent in gastric cancer tissues, GKN1 protein is treated as a potential biomarker for gastric cancer [34]. It is also found downregulated in placental tissue and cell [35]. Although current research focuses on the potential clinical use of GKN1 in the treatment of tumor, little is known about its expression and function in other organ systems or the significance of* GKN1 *polymorphisms in cancer. Our study firstly reports that polymorphismin* GKN1* is influence cisplatin based chemotherapy response in lung cancer patients. The SNPs from the other four genes* (ACTA2, AGPHD1, SEC14L5, *and* RBMS3) *included in this study did not reach any statistically significant association with lung cancer risks or cisplatin based chemotherapy response in our study population.

There are certain intrinsic limitations in our study and must be noted. The sample size was not as large as some other lung cancer association studies. We performed Bonferroni correction in our statistical analysis and found no statistical significant associations between* CRP* and* GPC5 *SNPs and lung cancer risk, neither in* GKN1* polymorphisms nor in response to cisplatin-based chemotherapy, which could be attributed to the relatively small sample size that may not satisfy all the seven independent hypotheses at the same time. Adjustments for multiple tests, like Bonferroni correction, are needed for medical association studies but may create more problems. The main weakness of Bonferroni correction is that the results depend on the number of other tests performed. True important differences may be deemed nonsignificant since the likelihood of type II errors is also increased [36]. Cumulatively, our findings provide evidence that polymorphisms in* C-reactive protein* and* Glypican 5* genes are associated with lung cancer risk, and* GKN1* determines chemotherapy response in Chinese population. We believe our results will encourage further studies to understand the function of these genes.

## Supplementary Material

Genotype frequencies of the seven tSNPs in lung cancer patients and controls.

## Figures and Tables

**Table 1:** Characteristics of patients and controls.

Characteristics	Lung cancer (*n* = 309)		Control (*n* = 310)	
Age (means ± SD, year)	58.2 ± 10.2		50.3 ± 8.1	
Sex				
Male	235	76.1	197	63.5
Female	74	23.9	113	36.5
Smoking status				
Never	94	30.4	188	60.6
Ever	215	69.6	122	39.4

	No.	%		

Histology				
Adenocarcinoma	110	35.6		
Squamous cell carcinoma	116	37.5		
Small-cell carcinoma	66	21.3		
Large-cell carcinoma	2	0.6		
Unspecified lung cancer	15	5.0		
Stage				
I	67	21.7		
II	52	16.8		
III	69	22.3		
IV	118	38.2		
Data uncertain	3	1.0		

**Table 2:** PCR primers.

SNP_ID	Forward primer	Reverse primer	UEP_SEQ
rs2808630	ACGTTGGATGGGGATGTAGGTTGAGCTAAT	ACGTTGGATGTAAGGCCAGAGGCTGTCTAC	tTTGCTTGCATCTTACTATAC
rs1926203	ACGTTGGATGAATCCACGTTACCTAAGCCC	ACGTTGGATGGGCTCTGATACCTGATTTGG	cgtgACCTAAGCCCAAATTATTAC
rs2352028	ACGTTGGATGATGACCCTGAACAGTAGTGG	ACGTTGGATGTGAAGGGTTTAACATGAAT	AGGGAAAGTCCATCTTTT
rs8034191	ACGTTGGATGCCACAAGTCCCCTTAGTTAC	ACGTTGGATGAGTGGTTAGAGCCCAATGTG	tTGTCAGGGCCTTTCT
rs9635542	ACGTTGGATGGAAGGTTGGTGGAAATTGCG	ACGTTGGATGTACAAACATGTACCCGGGTC	ttggcAATTGCGTGAGGAAAAG
rs4254535	ACGTTGGATGGAGACTGAAATAGAGTCTGC	ACGTTGGATGGATAGTTAGGACTCAACTGG	agAGAGTCTGCATGAAGGGAC
rs1530057	ACGTTGGATGTTCCATGAACAAAATGGAC	ACGTTGGATGTCAACATTATGGGCCACTCC	ggtggACAAAATGGACATGAACATGCAG

UEP_SEQ: unextended minisequencing primer.

**Table 3:** Candidate tSNPs.

SNP ID	Gene name	Chromosome position	Position	Allele	Minor allele	MAF (case)	MAF (control)	*P* value for HWE test	ORs	95% CI		*P* value from *x* ^2^	*P* value adj.^*^
rs2808630	*CRP *	1q23.2	159680868	C/T	C	0.135	0.191	0.982	0.66	0.48	0.91	0.010	0.070
rs1926203	*ACTA2 *	10q23.31	90727334	G/T	T	0.167	0.150	0.999	1.13	0.83	1.53	0.436	1
rs2352028	*GPC5 *	13q31.3	92445229	C/T	T	0.198	0.225	0.319	0.85	0.65	1.12	0.248	1
rs8034191	*AGPHD1 *	15q25.1	78806023	C/T	C	0.034	0.032	0.841	1.05	0.56	1.96	0.874	1
rs9635542	*SEC14L5 *	16p13.3	5001380	A/G	G	0.463	0.437	0.897	1.11	0.89	1.39	0.360	1
rs4254535	*GKN1 *	2p13.3	69198388	C/T	C	0.204	0.217	0.317	0.93	0.70	1.22	0.577	1
rs1530057	*RBMS3 *	3p24.1	29575463	A/C	A	0.065	0.078	0.788	0.82	0.53	1.27	0.368	1

^*^ *P* value was adjusted by Bonferroni correction.

**Table 4:** Relationship between rs2808630 of *CRP *and lung cancer risk (adjusted by gender and age).

Model	Genotype	Control (*N*, %)	Case (*N*, %)	OR (95% CI)	*P* value	*P* value adj.^*^	AIC	BIC
Codominant	T/T	189 (65.4%)	218 (75.4%)	1.00	0.100	0.500	710.2	732.0
	T/C	90 (31.1%)	64 (22.1%)	0.64 (0.43–0.97)				
	C/C	10 (3.5%)	7 (2.4%)	0.80 (0.28–2.30)				

Dominant	T/T	189 (65.4%)	218 (75.4%)	1.00	0.036	0.180	708.3	725.8
	T/C-C/C	100 (34.6%)	71 (24.6%)	0.66 (0.44–0.98)				

Recessive	T/T-T/C	279 (96.5%)	282 (97.6%)	1.00	0.850	1	712.7	730.1
	C/C	10 (3.5%)	7 (2.4%)	0.90 (0.32–2.58)				

Overdominant	T/T-C/C	199 (68.9%)	225 (77.8%)	1.00	0.037	0.185	708.3	725.8
	T/C	90 (31.1%)	64 (22.1%)	0.65 (0.43–0.98)				

Log-additive				0.72 (0.51–1.02)	0.061	0.305	709.2	726.6

AIC: Akaike's information criterion; BIC: Bayesian information criterion.

^*^ *P* value was adjusted by Bonferroni correction.

**Table 5:** rs2352028 of *GPC5* and gender cross-classification interaction.

Genotype	Female			Male			*P* value
	Control	Case	OR (95% CI)	Control	Case	OR (95% CI)	
C/C-T/T	82	47	1.00	110	163	1.80 (1.12–2.88)	0.019
C/T	31	27	1.42 (0.72–2.80)	87	71	0.98 (0.58–1.64)	

(*N* = 618, adjusted by age) under over-dominant model.

**Table 6:** Genotype and the allele frequencies of candidate genes in chemotherapy patients.

Genotype/allele	Responder		Nonresponder		OR^a^	95% CI		*P* value^a^	*P* value adj.^*^
	*N*	%	*N*	%					
rs2808630									
T/T	25	71.43	64	82.05	1.000				
T/C	9	25.71	10	12.82	0.434	0.158	1.194	0.142	0.994
C/C	1	2.86	4	5.13	1.652	0.166	14.670	0.791	1
T	59	84.29	138	88.46					
C	11	15.71	18	13.04	0.700	0.311	1.572	0.386	1
rs1926203									
G/G	23	65.71	55	70.51	1.000				
G/T	12	34.29	17	21.79	0.592	0.245	1.435	0.244	1
T/T	0	0	6	7.70	#	#	#	0.999	1
G	58	82.86	127	81.41					
T	12	17.14	29	18.59	1.104	0.526	2.316	0.989	1
rs2352028									
C/C	22	72.86	57	73.08	1.000				
C/T	13	37.14	21	26.92	0.623	0.267	1.457	0.221	1
T/T	0	0	0	0	—	—	—	—	
C	57	81.43	135	86.54					
T	13	18.57	21	13.46	0.682	0.320	1.455	0.269	1
rs8034191									
T/T	34	97.14	74	94.87	1.000				
T/C	1	2.86	4	5.13	1.838	0.198	17.068	0.477	1
C/C	0	0	0	0	—	—	—	—	
T	69	98.57	152	97.44					
C	1	1.43	4	2.56	1.816	0.199	16.547	0.485	1
rs9635542									
A/A	10	28.57	20	25.64	1.000				
G/A	17	48.57	37	47.44	1.088	0.420	2.819	0.866	1
G/G	8	22.86	21	26.92	1.312	0.431	3.996	0.809	1
A	38	54.29	76	48.72					
G	32	45.71	80	51.28	1.250	0.710	2.200	0.619	1
rs4254535									
T/T	28	80.00	46	58.97	1.000				
C/T	5	14.29	27	34.62	3.287	1.135	9.522	0.029	0.203
C/C	2	5.71	5	6.41	1.522	0.276	8.378	0.901	1
T	61	87.14	119	76.28					
C	9	12.86	37	23.72	2.107	0.955	4.649	0.109	0.763
rs1530057									
C/C	33	94.29	71	91.03	1.000				
C/A	2	5.71	7	95.51	1.627	0.320	8.260	0.659	1
A/A	0	0	0	0	—	—	—	—	
C	68	97.14	149	95.51					
A	2	2.86	7	4.49	1.597	0.323	7.891	0.671	1

*P* value ≤ 0.05 indicates statistical significance; OR: odds ratio; CI: confidence interval.

^a^Adjusted by age, gender, smoke status, histology, stage, and chemotherapy regimens.

^#^When a factor cell associated with the odds ratio is zero, extremely high odds ratios may occur, and it is the same with extremely low odds ratios. It is because the algorithm estimating the logistic coefficient (and hence also exp., the odds ratio) is unstable, failing to converge while attempting to move iteratively toward positive infinity (or negative infinity).

— Some of the mutated genotypes do not exist in the study subjects, so the relative statistics cannot be calculated.

^*^ *P* value was adjusted by Bonferroni correction.

## Abbreviations

tSNP: Tag single nucleotide polymorphism
*CRP*: * C-reactive protein* gene
*GPC5*: * Glypican 5* gene
*ACTA2*: * Actin*,* alpha 2*,* smooth muscle*,* aorta* gene
*AGPHD1*: * Aminoglycoside phosphotransferase domain containing 1* gene
*SEC14L5*: * SEC14-like 5* gene
*RBMS3*: * RNA binding motif*,* single stranded interacting protein 3* gene
*GKN1*: * Gastrokine 1* gene
LC: Lung cancer
MAF: Minor allele frequency
HWE: Hardy-Weinberg equilibrium
OR: Odds ratio
CI: Confidence intervals.
