# Supplementary material for: Polymorphisms in C-Reactive Protein and Glypican-5 Are Associated with Lung Cancer Risk and Gartrokine-1 Influences Cisplatin-Based Chemotherapy Response in a Chinese Han Population
Source: Dis Markers. 2015 Apr 27;2015:824304. doi: 10.1155/2015/824304 (PMC4426656; doi:10.1155/2015/824304)
Supplement: Supplementary file 1 — Genotype frequencies of the seven tSNPs in lung cancer patients and controls. [file 824304.f1.pdf]

Table S1. Genotype profiles in the study

SNP ID	A<br>Minor allele	B<br>Major allele	Lung cancer			Control		
			AA-FRE	AB-FRE	BB-FRE	AA-FRE	AB-FRE	BB-FRE
rs2808630	C	T	7	64	218	10	90	188
rs1926203	T	G	11	81	217	7	79	223
rs2352028	T	C	12	98	198	11	117	181
rs8034191	C	T	0	21	288	0	20	289
rs9635542	G	A	65	156	88	61	148	100
rs4254535	C	T	12	102	195	10	114	185
rs1530057	A	C	1	38	270	1	46	261
